# Urinary Fractional Clearance of Sodium in 8 Healthy Beagle Dogs Fed Normal, Low, or Ultralow Sodium Diets

**DOI:** 10.1155/2020/4106435

**Published:** 2020-02-18

**Authors:** R. G. Lobetti

**Affiliations:** Bryanston Veterinary Hospital, Box 67092, Bryanston, South Africa

## Abstract

The purpose of this study was to investigate in healthy adult dogs if there was a daily fluctuation in the FC_Na_, the role that dietary sodium intake played on the FC_Na_, and the role that feeding played on the obtained value for FC_Na_. Three different diets were used in a group of 8 healthy beagle dogs in a crossover design. The sodium content of the diets was normal (0.26%), low (0.18%), and ultralow (0.06%). Spot urine and blood samples were collected from which the urine and serum sodium and creatinine concentration were determined, and the FC_Na_ was calculated. The median FC_Na_ for the normal, low, and ultralow sodium diets was 0.5, 0.77, and 0.15, respectively. Individual dogs showed a daily variation in FC_Na,_ and samples which were collected shortly after eating showed the greatest variation. This study showed that in a group of healthy beagle dogs without obvious renal disease, the FC_Na_ could exceed 1 and that there was both an individual and daily variation in the FC_Na_. The greatest variation was seen whilst the dogs were fed the low and ultralow sodium diets and when the samples were collected shortly after eating. This study concluded that an FC_Na_ > 1% may not be indicative of acute tubular dysfunction in young dogs, and use of the FC_Na_ for assessing renal function in clinical cases should take into account the animal's diet, as well as the time the samples were taken in relation to feeding.

## 1. Introduction

Fractional clearance is defined as the fraction of the filtered solute that is not reclaimed as it passes through the renal tubular system. In order for the body homeostasis to be maintained, dietary intake needs to be matched by its excretion [1].

In order to maintain a stable plasma composition, the renal tubules either selectively reabsorb filtrate components or they secrete solutes delivered to them by the peritubular circulation [1]. The majority of extracellular sodium is actively reabsorbed in the proximal convoluted tubules [1]. Furthermore, sodium reabsorption takes place in the distal convoluted tubules secondary to active reabsorption of chloride ions and in the collecting ducts, the latter being controlled by the aldosterone [2].

Acute kidney injury (AKI) is a syndrome that is characterized by the sudden onset of impaired renal function resulting in azotaemia, increased fractional clearance of sodium (FC_Na_), and the presence of renal tubular epithelial cells and/or casts in the urine sediment [3–6]. Fractional excretion of electrolytes has been recently reevaluated in dogs with AKI as a readily available and cost-effective marker of tubular damage and kidney function [3, 5]. FC_Na_ was used as an early and accurate predictor of AKI in a population of dogs with naturally occurring heatstroke despite fluid resuscitation [5]. Although it is generally accepted that a FC_Na_ > 1% is indicative of acute tubular dysfunction [4], an incidental finding in two studies showed that healthy young dogs often had a FC_Na_ > 1% in the absence of obvious signs of renal dysfunction [7, 8]. Another study showed that FC_Na_ was not different between volume-responsive AKI and control dogs [6]. These studies attest to the paucity of data in the veterinary literature and the lack of inclusion of healthy control dogs.

It is possible that an FC_Na_ > 1% may not always be indicative of acute tubular dysfunction and that values of this magnitude could merely reflect an increased salt intake by the animal. In addition, the FC_Na_ can also be influenced by the administration of sodium-containing fluids, which can increase the FC_Na_ and may negate the usefulness in using FC_Na_ as a diagnostic test [3, 5].

Feeding has been shown to affect the quantity of sodium that is excreted in the urine of clinically healthy dogs [9]. However, none of the dogs in that study showed an FC_Na_ > 1%. In one study in dogs with chronic kidney disease (CKD), the FC_Na_ was proportional to the dietary sodium intake [10]. In that study, 3 of the diets were high in sodium (1.17%, 0.95%, and 0.58%) and 1 had a normal content (0.25%) and all dogs had an FC_Na_ < 1%. Another study showed that the FC_Na_ was higher in dogs with CKD compared to healthy dogs [11]. In the same study, healthy dogs fed either a normal (0.23%) or high-sodium (0.41%) diet did not have an FC_Na_ > 1% [11]. It has been alluded that prior to determining the fractional clearance of electrolytes, dogs should be fed a consistent diet for approximately 1 week before submission of samples [4].

Although urine collection over a 24-hour period is most accurate for determining the fractional clearance of electrolytes, spot samples of simultaneously collected urine and plasma provide clinically reasonable approximations of total daily excretion despite some variability [4]. Correlation has been shown between spot and 24-hour collection determinations [12, 13].

The purpose of this study was to investigate if there was a daily fluctuation in the FC_Na_, the role that dietary sodium intake plays on the FC_Na_, the role that feeding plays on the obtained value for FC_Na_, and whether or not the time of sample collection in relation to feeding can influence FC_Na_. The main hypothesis of the study was that an elevated FC_Na_ may not be indicative of acute tubular dysfunction if the animal was fed a high-sodium diet.

## 2. Materials and Methods

### 2.1. Animals

Eight beagles (3 males and 5 females) from the Onderstepoort Animal Teaching Unit were used in the study. Seven of the dogs were 6 years of age and one 4 years. Prior to the study, all the dogs were screened for preexisting renal disease by means of full urine analysis and serum biochemistry (urea, creatinine, calcium, phosphate, sodium, and potassium). Fractional clearance of sodium was also determined in all dogs. The dogs were kept in an appropriate animal management facility, and the study was approved by the Animal Research and Ethic Committee of the Faculty of Veterinary Science, University of Pretoria. The physical and biochemical examinations performed before the study confirmed that all dogs were healthy.

### 2.2. Experimental Procedures

The effect of 3 diets on fractional excretion of sodium was assessed: diet 1 had a normal sodium content (Hills adult maintenance®, 0.26% sodium), diet 2 moderately reduced (Hills k/d®, 0.18% sodium), and diet 3 severely reduced (Hills h/d®, 0.06% sodium). All dogs and diets were used in a crossover study with a 2-week period being allowed for acclimatisation of the new diet before the samples were collected.

### 2.3. Data Collection

The dogs were housed in their normal environment, fed twice a day, and had access to *ad-lib* water. The sodium content in the dogs' drinking water was quantified to be less than 10 mg/l. During the first 2 weeks, no samples were collected from the animals. From the third week onwards, blood and urine samples were collected daily. In the third week, samples were collected after the animals had been fasted for approximately 12–14 hours and in the fourth week samples were collected approximately 2-3 hours after eating. All samples were collected at approximately 10.00 am. Serum and urine creatinine were determined on a Technicon RA 1000 system (Technicon Instruments Corporation, Tarrytown, USA). Serum and urine sodium were determined using an ion selective analyzer (Rapidlab™ 348 pH/Blood gas analyzer, Chiron Diagnostics, Essex, UK).

The fractional clearance of sodium was calculated using the following formula:

(1) $$\begin{array}{c} \frac{\text{urine sodium}\left(\text{mmol}/\text{l}\right)}{\text{serum sodium}\left(\text{mmol}/\text{l}\right)}\times \frac{\text{serum creatinine}\left(\mu \text{mmol}/\text{l}\right)}{\text{urine creatinine}\left(\mu \text{mmol}/\text{l}\right)}\times 100. \end{array}$$

### 2.4. Statistical Analysis

Data were tabulated in a spreadsheet program (Excel, Microsoft Corporation, USA). Statistical analysis was performed with the aid of a statistical software package (Sigma Stat, Jandal Corporation, USA), and the generated data were graphically depicted with the aid of a graphic software package (Sigma Plot, Jandal Corporation, USA). Descriptive statistics were used to describe the data with the Kruskal–Wallis one-way analysis of variance on ranks, and the Wilcoxon signed rank test was used to test the statistical difference between groups. The level of significance was set at *p* < 0.05.

## 3. Results

**Figure 1:**
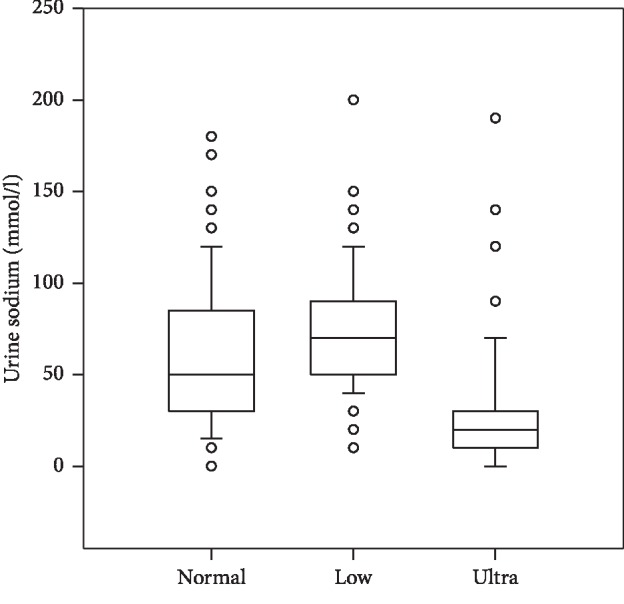
Urine sodium concentrations in dogs fed normal (0.26%), low (0.18%), and ultralow (0.06%) sodium diets. Data are shown as median (horizontal line within the box), 25th and 75th percentiles (horizontal ends of boxes), and 10th and 90th percentiles (T-bars). Open circles represent outliers. There was a significant difference between the 3 groups (*p* < 0.05).

**Figure 2:**
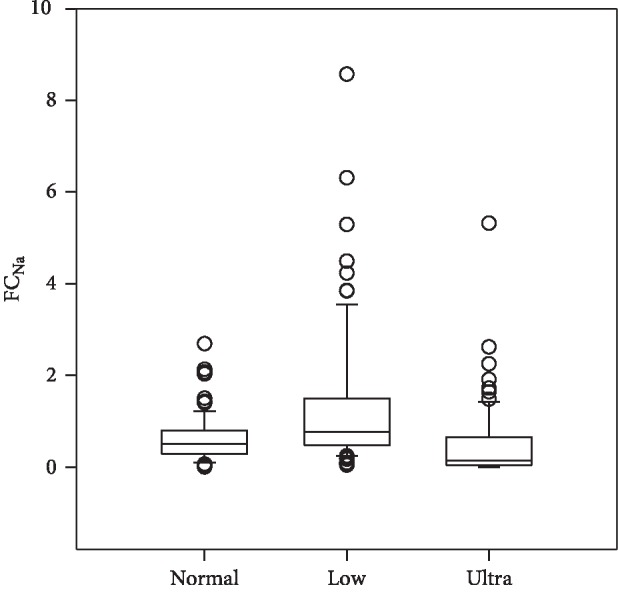
Fractional clearance of sodium in dogs fed normal (0.26%), low (0.18%), and ultralow (0.06%) sodium diets. Data are shown as median (horizontal line within box), 25th and 75th percentiles (horizontal ends of boxes), and 10th and 90th percentiles (T-bars). Open circles represent outliers. There was a significant difference between the 3 groups (*p* < 0.05).

**Figure 3:**
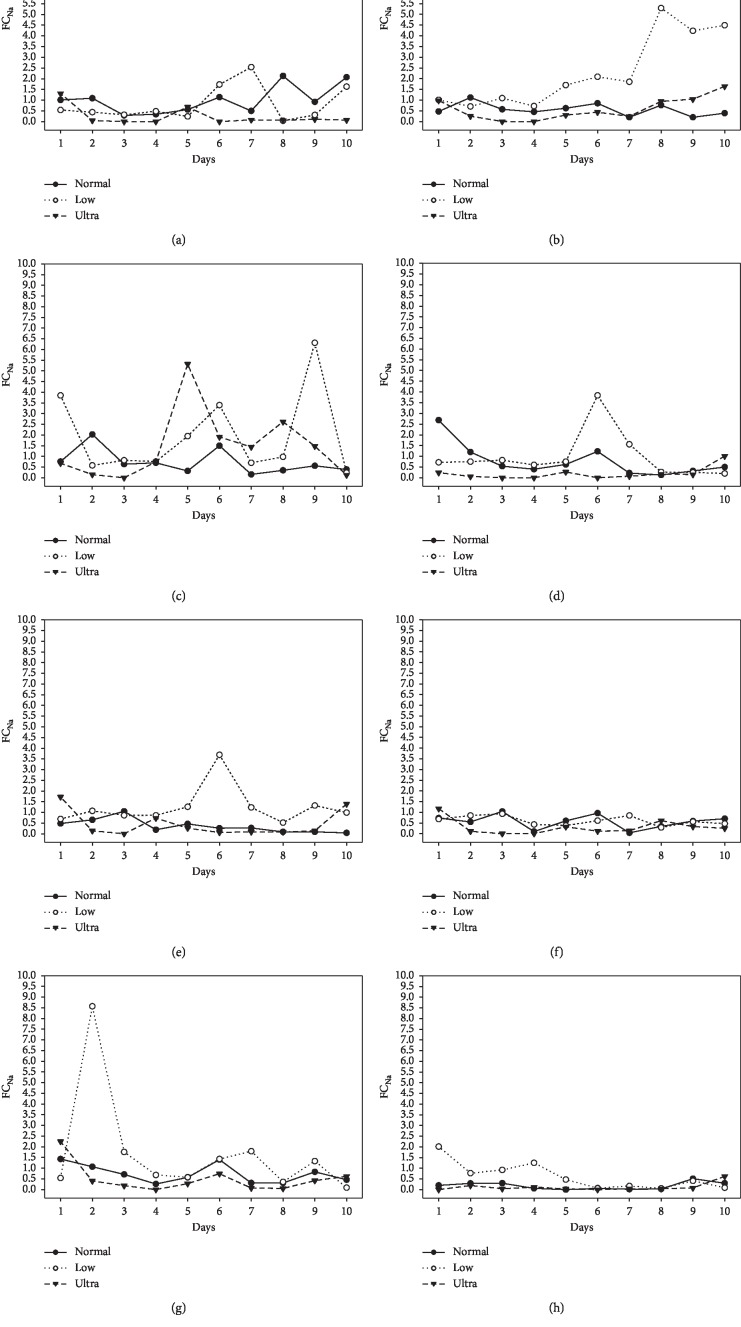
Individual fractional clearance of sodium over the duration of the study. (a) Dog 1. (b) Dog 2. (c) Dog 3. (d) Dog 4. (e) Dog 5. (f) Dog 6. (g) Dog 7. (h) Dog 8.

The results are tabulated in Table 1 and graphically depicted in Figures 1–3. Median FC_Na_ for the normal, low, and ultralow sodium diets was 0.5, 0.77, and 0.15, respectively, in which there was a statistical difference between the 3 groups. The FC_Na_ range for the 3 groups was 0 to 8.57. Urine sodium values mirrored the FC_Na_ results and in that there was a statistical difference between all 3 groups. There was no statistical difference with the serum sodium values between the 3 groups.

Individual dogs showed a daily variation in FC_Na,_ and samples collected shortly after eating showed the greatest variation.

## 4. Discussion

This study showed that in a group of healthy adult beagle dogs with no evidence of renal dysfunction, FC_Na_ could exceed a value of 1% and that there was both an individual and daily variation. The greatest variation was seen whilst the dogs were fed the low and ultralow sodium diets, but some dogs on the normal sodium diet had sporadic FC_Na_ values > 1%. This finding of an FC_Na_ > 1% supports the incidental observation noted in two other studies that healthy young dogs can have FC_Na_ values > 1% with no obvious renal injury [7, 8].

This study showed that if samples were collected after food had been withheld for a period of time (12–14 hours), there was a tendency for the FC_Na_ to be less than 1% although there was an individual variation. This finding is in agreement with a previous study that showed that in healthy beagle dogs where food was withheld had a significant decrease in urinary excretion of sodium [2]. The dogs in that study were fed a reduced sodium diet (0.18%). This finding can be expected as the kidneys primarily eliminate sodium.

In another study in dogs that were fed a normal (0.23%) and a high-sodium (0.41%) diet, FC_Na_ never exceeded 1% [11]. In this current study, individual dogs fed either the low (0.18%) or ultralow (0.06%) sodium diet had sporadic FC_Na_ > 1% although the median values for all 3 diets were <1%. The highest value recorded for the FC_Na_ with the normal, low, or ultralow sodium diets, where 2.69, 8.57, and 5.32, respectively.

This study utilised a spot urine sample to determine the FC_Na_, which can be influenced by the circadian variation in the urinary excretion of sodium [11], which was evident in this study by both daily and individual variation. Previous studies have shown correlation between spot and 24-hour collection determinations of FC_Na_ [12, 13]. In a clinical setting, the spot test is more practical than the other 2 collection methods.

This study concluded that an FC_Na_ > 1% may not be indicative of acute tubular dysfunction in young dogs, and use of the FC_Na_ for assessing renal function in clinical cases should take into account the animal's diet as well as the time the samples were taken in relation to feeding.

## Figures and Tables

**Table 1:** Fractional clearance of sodium (FCNa), serum sodium, and urine sodium concentrations in dogs fed normal (0.26%), low (0.18%), and ultralow (0.06%) sodium diets.

	Diets	Mean	Median	SD	Range
FC_Na_ (%)	Normal	0.62	0.5	0.52	0–2.69
	Low	1.29	0.77	1.48	0.04–8.57
	Ultra	0.47	0.15	0.79	0–5.32

Serum sodium (mmol/l)	Normal	148.06	148	1.50	144–151
	Low	148.93	149	2.06	141–154
	Ultra	148.12	148	2.41	141–155

Urine sodium (mmol/l)	Normal	61.85	50	41.03	0–180
	Low	74	70	33.63	10–200
	Ultra	29	20	35.53	0–190

## Data Availability

The raw data used to support the findings of this study are available from the corresponding author upon request.
